# Flavonoids from *Apios americana* Medikus Leaves Protect RAW264.7 Cells against Inflammation via Inhibition of MAPKs, Akt-mTOR Pathways, and Nfr2 Activation

**DOI:** 10.1155/2019/1563024

**Published:** 2019-12-13

**Authors:** Qiang Chu, Xin Yu, Ruoyi Jia, Yaxuan Wang, Yiru Zhang, Shuang Zhang, Yangyang Liu, Yonglu Li, Wen Chen, Xiang Ye, Xiaodong Zheng

**Affiliations:** ^1^Department of Food Science and Nutrition, National Engineering Laboratory of Intelligent Food Technology and Equipment, National-Local Joint Engineering Laboratory of Intelligent Food Technology and Equipment, Key Laboratory for Agro-Products Postharvest Handling of Ministry of Agriculture, Zhejiang Key Laboratory for Agro-Food Processing, Fuli Institute of Food Science, Zhejiang University, Hangzhou 310058, China; ^2^State Key Laboratory of Silicon Materials, School of Materials Science and Engineering, Zhejiang University, Hangzhou 310027, China; ^3^College of Environmental and Resource Sciences, Zhejiang University, Hangzhou 310058, China

## Abstract

*Apios americana* Medikus was once widely accepted as staple food in India for a long time, and the tuber of which possesses high nutrients. During the past decades, most of the research has focused on the biological activity in the tubers of *Apios americana* Medikus whereas the leaves were ignored. In this study, the *Apios americana* Medikus leaf extract (ALE) was obtained and seven compounds were identified. LPS-induced RAW264.7 cells were used to study the anti-inflammation activity of ALE. As expected, ALE reduced the secretion of nitric oxide (NO) and inflammatory cytokines via inhibition of NF-*κ*B and MAPK signaling together with activation of Nrf2-Keap1 and FOXO pathways, as well as alleviating the oxidative stress and mitochondrial dysfunction. In addition, ALE could activate HMGB1-Beclin1 and Sirt1-FoxO1 pathways and inhibit the Akt-mTOR signaling pathway to activate autophagy, protecting RAW264.7 cells from inflammation. In summary, our results suggested that ALE might help activate the anti-inflammation system, resulting in the prevention of LPS-induced damage in RAW264.7 cells.

## 1. Introduction

Inflammation is a natural defensive response of the organisms to stimulation. Many factors could induce inflammation, which are involved in pathogens, chemical immune responses, damaged cells, irritants, etc. Excessive inflammation response would lead to disorder in organisms together with the overproduction of oxygen free radicals, eventually breaking the dynamic balance [1], which tends to cause iseases like fever, asthma, psoriasis, arthritis, and even cancer [2]. Meanwhile, macrophages play a vital part during the process of inflammation and many cytokines are generated, among which interleukin-1 (IL-1), interleukin-6 (IL-6), and tumor necrosis factor *α* (TNF-*α*) could further promote inflammation. Therefore, the key to treat inflammation is to suppress the aberrant macrophage activation.

Multiple studies have demonstrated that lipopolysaccharide (LPS) induces the generation of inflammatory cytokines via NF-*κ*B and MAPK pathways [3]. RAW264.7 cells induced by LPS have widely been accepted as the classic inflammation model. Macrophages will produce a huge number of inflammatory cytokines like NO, TNF-*α*, IL-*β*, and COX under LPS conditions [4]. However, the incessant inflammation will cause the overproduction of reactive oxygen/nitrogen species including O_2_^·−^, H_2_O_2_, OH^−^, ONOO^·^, and HOONO, leading to DNA damage, mitochondrial dysfunction, and cell death. The mitochondrion is the site where reactive oxygen/nitrogen species generate; meanwhile, mitochondrial dysfunction is closely connected with the overproduction reactive oxygen/nitrogen species [5]. In addition, as a self-protective mechanism of cells, autophagy could lead to mitochondrial transformation and the decomposition of impaired mitochondria. It has been proven that cell autophagy facilitates in scavenging reactive oxygen species, maintaining mitochondrial function, and attenuating inflammation [6, 7]. The balance between inflammation and autophagy is associated with many pathways such as the FoxO1 pathway, Akt-mTOR pathway, and HMGB1 pathway [8, 9].

Existing anti-inflammatory drugs always produce a side effect on the gastrointestinal tract and cardiovascular systems, suggesting that seeking other safe, efficient, and noncytotoxic anti-inflammatory drugs is necessary [10]. In the last few decades, more and more scientists have paid attention to the functional factors derived from plants for their favorable function of regulating the immune system and inflammation. *Apios americana* Medikus is a nitrogen-fixing legume native to North America, and the tubers of which are abundant in fatty acid, lipids, carbohydrate, and amino acids [10, 11]. However, most of studies on *Apios americana* Medikus focused on the biological activities of its tubers, and the leaves were ignored. In this study, flavonoids were extracted from dried *Apios americana* Medikus leaves and the main components were identified by liquid chromatography-tandem mass spectrometry (LC-MS/MS) analysis. Subsequently, we aimed to investigate the potential protective effect of ALE against LPS-induced inflammation in RAW264.7 cells and explored the underlying mechanism.

## 2. Materials and Methods

### 2.1. Materials and Reagents

RAW264.7 cells were purchased from the Wuhan Cell Bank of Chinese Academy of Sciences. Absolute ethyl alcohol, methanol, ethyl acetate, potassium bromide, formic acid, glucan, dimethyl sulfoxide, hydrochloric acid, and acetonitrile were obtained from Aladdin (Shanghai, China). Trypsin and MTT were purchased from Sigma-Aldrich (St. Louis, MO, USA). Fetal bovine serum and DMEM high glucose medium were obtained from Jinnuo Biotechnology (Hangzhou, China). 2′,7′-Dichlorofluorescin diacetate (DCFH-DA), 3-amino,4-aminomethyl-2′,7′-difluorescein diacetate (DAF-FMDA), dihydroethidium (DHE), naphthalene-2,3-dicarboxaldehyde (NDA), Nonyl Acridine Orange (NAO), Rhodamine 123, and Hoechst 33258 were purchased from Yusheng Biotechnology (Shanghai, China). ELISA kits were obtained from Jiyinmie Biotechnology (Wuhan, China). Primary antibodies were purchased from Abcam (Shanghai, China). NO assay kit, *β*-actin, the ECL Western blotting system, WB/IP lysis buffer, and BCA protein assay kit were purchased from Beyotime Biotechnology (Jiangsu, China). Other chemicals were of reagent grade from Aladdin (Shanghai, China).

### 2.2. Purification and Identification of ALE

Fresh *Apios* leaves were weighed, extracted, and mixed with water at a 1 : 10 ratio and treated by ultrasonic extraction at room temperature for 2 h. Then, the extract was centrifuged at 4000 r/min for 30 min, and centrifugation is repeated 3 times. The extract (1 g) was dissolved in 50 mL purified water, applied to a DM301 microporous resin column (Ø3.2 × 60 cm), washed with 1 L of water, and eluted at a flow rate of 1.0 mL/min with 20%, 40%, 60%, and 80% ethanol, respectively. After collection, the darker part of the eluent was merged, freeze-dried, and purified as ALE. ALE was stored at -80°C for subsequent experiments. The freeze-dried extract samples were dissolved in ultrapure water and prepared into a solution of 0.5 mg/mL. The main components were analyzed by LC-MS/MS after filtration with a 0.22-micron filter membrane. An Ultra Performance Liquid Chromatography (UPLC) system (Waters, Milford, MA, USA) was used for qualitative analysis. The flow rate was set at 0.8 mL/min and the temperature was maintained at 40°C. Solvent A was 15% (*v*/*v*) acetonitrile+0.05 mol/L phosphate buffer (KH_2_PO_4_-NaOH, pH 6.9), and solvent B was 40% (*v*/*v*) acetonitrile+0.05 mol/L phosphate buffer. The solvent gradient of phase A was as follows: 0–15 min, 0–95% B; 15–21 min, 95–85% B; 21–22 min, 85–72% B; 22–24 min, 72–60% B; 24–27 min, 60–40% B; and 27–30 min, 40–95% B.

### 2.3. Cell Culture and Treatment

RAW264.7 cells were cultured in DMEM containing 10% fetal bovine serum, at 37°C, in a 5% CO_2_ incubator. After 80% confluence, the cells were inoculated with ALE of different concentrations for 24 hours, and then, 250 ng/mL LPS was added for a total of 12 hours. Cells without LPS and ALE were used as a negative control group. Cells treated with LPS alone were used as a model group.

### 2.4. MTT Assays

RAW264.7 cells were seeded into a 96-well plate for 24 h. After treatments with ALE or LPS at different concentrations for 24 h, 100 *μ*L (0.5 mg/mL) 3-(4,5 dimethylthazol-2-yl)-2,5-diphenyltetrazolium ammonium bromide (MTT) was added into each well, which was diluted with DMEM. After incubation at 37°C for 4 hours, formazan was precipitated and dissolved in 150 *μ*L dimethyl sulfoxide (DMSO). The absorbance was measured at 570 nm with a spectrophotometer [12–14].

### 2.5. Nitrite Analysis

Nitric oxide levels were detected by the Griess reagent method. After treatment, RAW264.7 cell lysate and supernatant were harvested and the products from NO reactions were measured according to the instruction of the NO assay kit. The level of intracellular NO was quantified by total protein concentration. The protein concentration in the cell lysates was detected with the BCA protein concentration assay kit.

### 2.6. Detection of the Secretion of Cytokines

After treatment, the supernatant was collected, and the concentrations of TNF-*α*, IFN-*γ*, IL-1*β*, IL-6, and IL-10 in the medium were determined according to ELISA kit instructions.

### 2.7. Fluorescent Staining

Fluorescent probes were used to detect intracellular ROS (DCFH, 10 mM), NO (DAF-FMDA, 5 *μ*M), superoxide radical generation (DHE, 10 *μ*M), GSH consumption (NDA, 50 *μ*M), DNA damage (Hoechst 33342, 10 *μ*M), mitochondrial membrane potential alteration (RH123, 5 *μ*M), mitochondrial membrane lipid peroxidation (NAO, 20 *μ*M), and the number of mitochondria (MitoTracker Green, 20 *μ*M). After treatment, the cells were incubated in phenol-free red medium containing different probes for 30 minutes. Then, the cells were washed with PBS 2 times and analyzed with a fluorescence microscope. The results were expressed as fluorescence intensity calculated by the image analysis software Image-Pro Plus 6.0.

### 2.8. Transmission Electron Microscopy (TEM)

After grouping and treatment, cells in each group were collected and centrifuged at 1000 rpm for 5 min. Then, the ultrastructural changes were observed with a Hitachi H-7650 transmission electron microscope (Hitachi, Japan) operated at 15000 and 30000 magnifications after the process of fixation, rinsing, dehydration, embedding, ultrathin sectioning, and staining.

### 2.9. Western Blot

Cells were collected and lysed in a WB/IP lysis buffer. The lysate was centrifuged at 4°C and 12000 g for 10 min. The protein concentration was measured and leveled with the BCA protein assay kit. Equal amounts of protein (40 mg) are separated on SDS-PAGE and transferred to PVDF membranes. Then, the membrane was blocked with appropriate 5% nonfat milk and probed with 4 mL primary antibodies at 4°C overnight. The primary antibodies included iNOS, COX2, NF-*κ*B, Keap1, Nrf2, p-JNK, JNK, p-ERK, ERK, p-p38, p38, Rab5, Rab7, Atg4, Atg5, Beclin1, LC3B, p62, HMGB1, p-FOXO1, FOXO1, p-Akt, Akt, p-mTOR, mTOR, and *β*-actin antibodies (Abcam). Horseradish peroxidase- (HRP-) conjugated secondary antibodies were probed to the membrane for 2 h at room temperature. The ECL luminescent liquid was added to the PVDF film. The protein bands were exposed to an Invitrogen iBright 1500 gel imaging system, and the densitometry of them was analyzed by the ImageJ software while *β*-actin was used as an internal reference [12].

### 2.10. Data Statistics and Analysis

In the study, every experiment contained at least 3 biological repetitions and every biological repetition was conducted with at least 3 technical repeats. Fluorescence images were analyzed by Image-Pro Plus for average optical density. Western blot results were quantified by ImageJ. Experimental data were expressed as the means and SD and analyzed by SPSS (Statistical Product and Service Solutions) 19.0 statistical software. Variance analysis was performed by one-way analysis of variance (ANOVA) followed by Duncan's multiple range test by Duncan's multiple comparisons. *p* < 0.05 was considered significant, and data were plotted with GraphPad Prism 5 [15, 16].

## 3. Results

### 3.1. Composition and Identification of ALE

**Figure 1:**
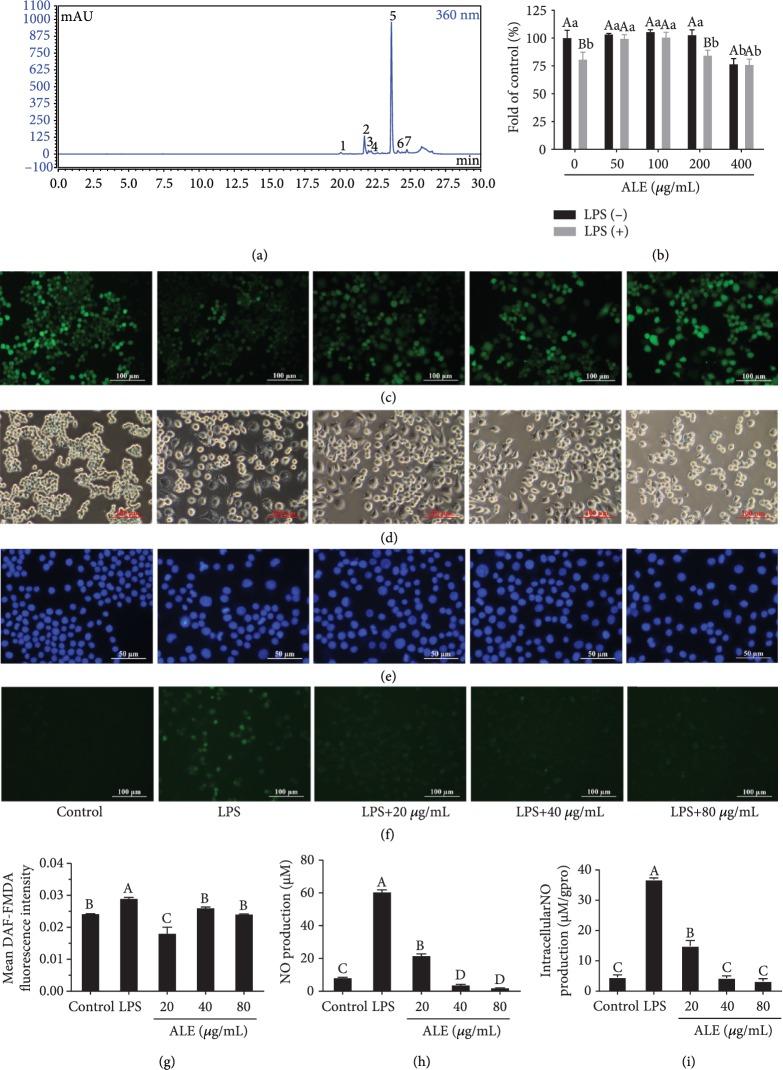
HPLC elution profiles of compounds in *Apios americana* Medik leaves and effects of ALE on LPS-induced cytotoxicity and inflammation in RAW264.7 cells. (a) The liquid chromatography profile of ALE in *Apios americana* Medik leaves. The peak numbers were labeled according to the retention times. (b) RAW264.7 cell viability was measured by the MTT method after treatment with ALE at different concentrations for 24 h with/without LPS inducement (*n* = 6). (c) Cell proliferation assay via BeyoClick™ EdU Cell Proliferation Kit with Alexa Fluor 488. (d) Morphological changes of RAW264.7 cells after ALE treatment (*n* = 3). (e, f) Cell apoptosis, NO staining with Hoechst 33258, and DAF-FMDA in the presence of LPS and ALE (*n* = 3). (g) The quantitative data of (f). (h) NO production in cell culture medium according to the Griess reagent method (*n* = 4). (i) Intracellular concentration of NO according to the Griess reagent method quantified by total protein concentration (*n* = 4). ALE-treated cells were inoculated in different concentrations of ALE for 24 hours, and then, 250 ng/mL LPS was added for a total of 12 hours. Cells without LPS and ALE were used as a negative control group. Cells treated with LPS alone were used as a model group. Images were captured with a fluorescence microscope in the same settings. All the fluorescence images were quantified in the whole field with the background removed and represented by normalized fluorescence (*y*-axes) via Image-Pro Plus 6.0 (*n* = 3). Significance analysis was carried out according to the one-way ANOVA test, and different letters in figures mean statistically significant differences among the groups (A, B, C, and D were labeled from large to small and the same letters in columns mean statistical insignificance; otherwise, they mean statistical significance (*p* < 0.05)).

According to the HPLC-MS/MS (Figure 1(a)) result, there are seven main components in ALE (Table 1): vicenin-2 (peak 1) [17], schaftoside (peak 2) [18], baimaside (peak 3), apigenin (6-C-*α*-L-arabinopyranosyl)-8-C-*β*-D-glucopyranoside (peak 4) [12], vitexin (peak 5), isquercetin (peak 6), and 6-C-*β*-D-glucopyranosyl-7-O-methylluteolin (peak 7). The highest content of the component in ALE was vitexin, followed by schaftoside, and the proportion of other components was low. Besides, Table 1 showed the peak identification, retention times, MS/MS date, and compound identification of the components.

### 3.2. ALE Attenuates LPS-Induced Cytotoxicity and Inflammation in RAW264.7 Cells

LPS was applied to induce the inflammation of RAW264.7. The results in Figures 1(b)–1(d) showed that LPS stimulation induced the decline in cell proliferation activity and led to morphological changes of RAW264.7 cells. However, the pretreatment of ALE at different concentrations could reverse the situation induced by LPS (Figures 1(b) and 1(c)). It could be observed that ALE at concentrations below 100 *μ*g/mL had no toxicity on RAW264.7 cells and could alleviate the cytotoxicity induced by LPS. Then, we used the Hoechst 33258 fluorescence probe to measure the protective effect of ALE [19]. Compared with the LPS-induced group, the number of bright blue points in the ALE-treated group was significantly reduced, and the LPS-induced chromatin condensation was attenuated, suggesting that ALE had a positive effect on LPS-induced cell apoptosis (Figure 1(e)). Besides, we used the DAF-FMDA fluorescence probe to examine intracellular NO levels (Figure 1(f)). The ALE-treated group significantly reduced the content of intracellular NO (Figure 1(g)). In addition, Figures 1(h) and 1(i) show that the stimulation of LPS could significantly increase the level of NO in cells and in culture medium, while ALE inhibited such increases (Figures 1(h) and 1(i)). Herein, the above results showed that ALE helped relieve inflammation on LPS-induced RAW264.7 cells.

### 3.3. ALE Decreases LPS-Induced Overproduction of Inflammatory Cytokines in RAW264.7 Cells

**Figure 2:**
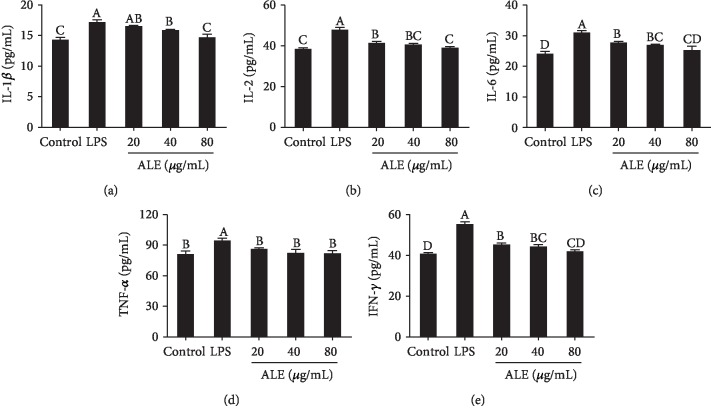
Effects of ALE on LPS-induced secretion of inflammatory factors in RAW264.7 cells. (a–e) IL-1*β*, IL-2, IL-6, TNF-*α*, and IFN production in cell culture medium according to the ELISA method (*n* = 4). ALE-treated cells were inoculated in different concentrations of ALE for 24 hours, and then, 250 ng/mL LPS was added for a total of 12 hours. Cells without LPS and ALE were used as a negative control group. Cells treated with LPS alone were used as a model group. Images were captured with a fluorescence microscope in the same settings. Significance analysis was carried out according to the one-way ANOVA test, and different letters in figures mean statistically significant differences among the groups (A, B, C, and D were labeled from large to small and the same letters in columns mean statistical insignificance; otherwise, they mean statistical significance (*p* < 0.05)).

Cytokines such as IL-1*β*, IL-2, IL-6, TNF-*α*, and IFN-*γ* are involved in the inflammation process [20]. Therefore, we further detected the production of inflammatory cytokines in cell culture medium. The results showed that LPS stimulated the elevation of inflammatory cytokines. However, the treatment of ALE at different concentrations suppressed the secretion of inflammatory cytokines in RAW264.7 cells (Figure 2), suggesting the alleviation of inflammation induced by LPS.

### 3.4. ALE Ameliorates LPS-Induced Oxidative Damage in RAW264.7 Cells

**Figure 3:**
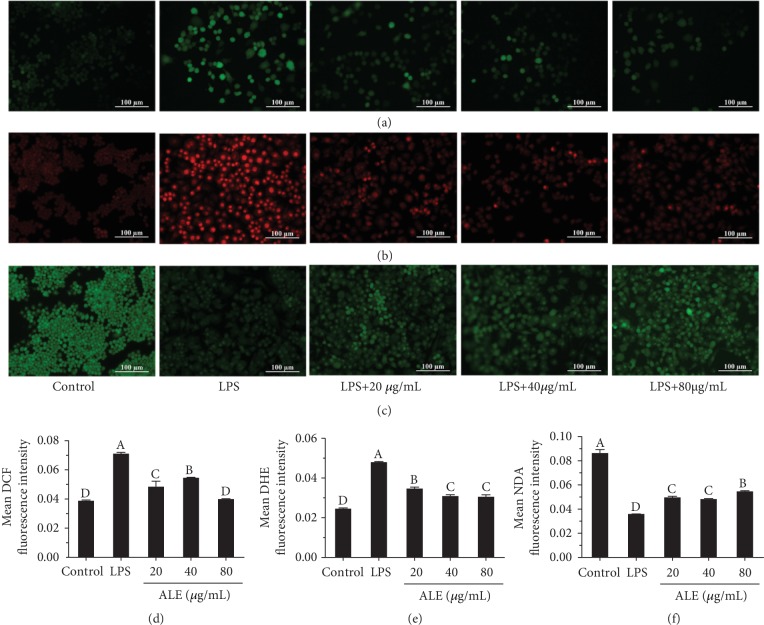
Effects of ALE on LPS-induced oxidative damage in RAW264.7 cells (*n* = 3). (a) DCFH-DA staining for ROS. (b) DHE staining for O_2_^·−^. (c) NDA staining for GSH. (d) The quantitative data of (a). (e) The quantitative data of (b). (f) The quantitative data of (c). ALE-treated cells were inoculated in different concentrations of ALE for 24 hours, and then, 250 ng/mL LPS was added for a total of 12 hours. Cells without LPS and ALE were used as a negative control group. Cells treated with LPS alone were used as a model group. Images were captured with a fluorescence microscope in the same settings. All the fluorescence images were quantified in the whole field with the background removed and represented by normalized fluorescence (*y*-axes) via Image-Pro Plus 6.0 (*n* = 3). Significance analysis was carried out according to the one-way ANOVA test, and different letters in figures mean statistically significant differences among the groups (A, B, C, and D were labeled from large to small and the same letters in columns mean statistical insignificance; otherwise, they mean statistical significance (*p* < 0.05)).

LPS-induced inflammation is accompanied by oxidative stress [21]. Natural antioxidants, such as flavonoids, have potential ability to reduce oxidative stress. As shown in Figure 3, LPS significantly increased the levels of intracellular ROS (Figures 3(a) and 3(d)) and O_2_^·−^ contents (Figures 3(a) and 3(e)) and decreased the level of glutathione (GSH) (Figures 3(c) and 3(f)). ALE treatment significantly inhibited the formation of ROS and O_2_^·−^ and recover the level of GSH content, indicating the relief of oxidative stress induced by LPS. Studies have reported that natural products could inhibit the production of ROS and O_2_^·−^ and increase the production of GSH [21]. Meanwhile, our previous studies have shown that extracts from the flowers and leaves of *Apios americana* Medikus could also increase the levels of GSH in cells, which is beneficial to HepG2 cells' resistance to H_2_O_2_-induced oxidative stress [22].

### 3.5. ALE Attenuates LPS-Induced Mitochondrial Dysfunction in RAW264.7 Cells

**Figure 4:**
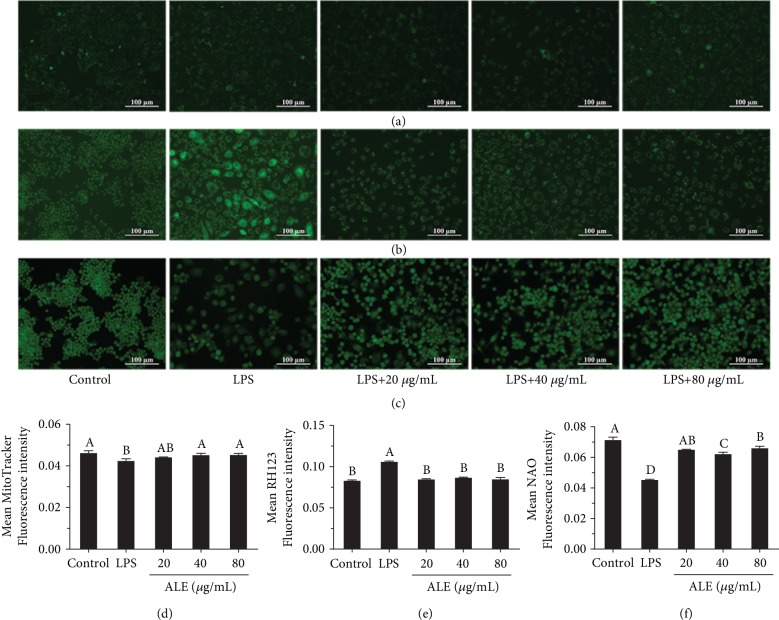
Effect of ALE on LPS-induced mitochondrial dysfunction in RAW264.7 cells (*n* = 3). (a–c) Mitochondria, mitochondrial membrane potential, and mitochondrial membrane lipid peroxidation alterations of RAW164.7 cells in the presence of LPS and ALE under different treatments which were incubated with MitoTracker, RH123, and NAO. (d–f) The quantitative data of (a), (b), and (c). Images were captured with a fluorescence microscope in the same settings. All the fluorescence images were quantified in the whole field with the background removed and represented by normalized fluorescence (*y*-axes) via Image-Pro Plus 6.0 (*n* = 3). Significance analysis was carried out according to the one-way ANOVA test, and different letters in figures mean statistically significant differences among the groups (A, B, C, and D were labeled from large to small and the same letters in columns mean statistical insignificance; otherwise, they mean statistical significance (*p* < 0.05)).

**Figure 5:**
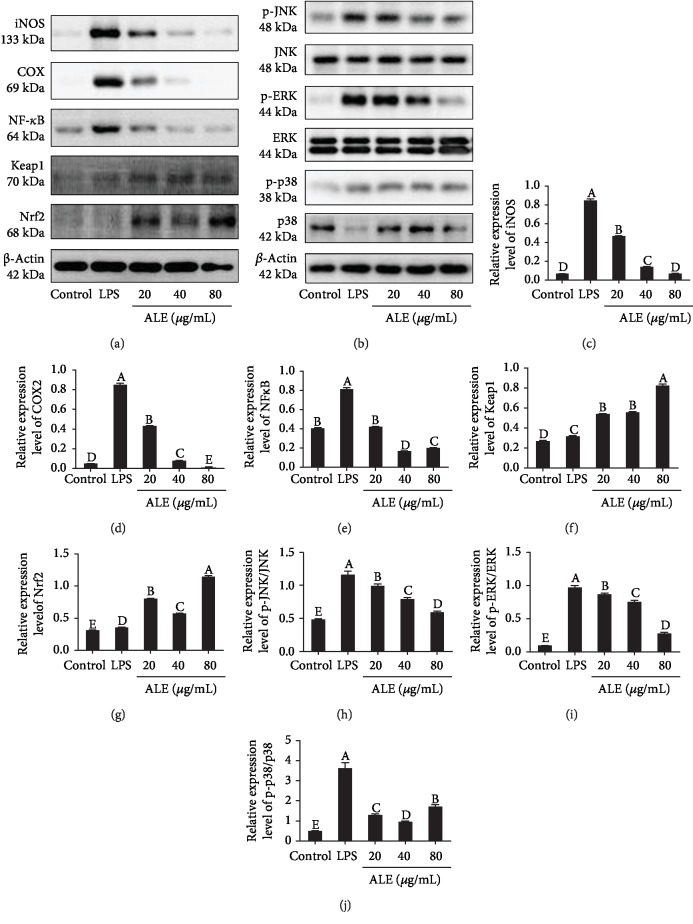
ALE treatment altered expressions of proteins associated with inflammation and oxidative stress in RAW264.7 cells. (a) Western blot bands of iNOS, COX2, NF-*κ*B, and Nrf2-Keap1 (*n* = 3). (b) Western blot bands of MAPKs. (c–j) Relative expressions of iNOS, COX2, NF-*κ*B, Keap1, Nrf2, p-JNK/JNK, p-ERK/ERK, and p-p38/p38. The images were quantified by the ImageJ software, and the intensity of bands was corrected with *β*-actin (*n* = 3); vertical lines in the histogram represent SDs of three replicates. Significance analysis was carried out according to the one-way ANOVA test, and different letters mean statistically significant differences among the groups (A, B, C, D, and E were labeled from large to small and the same letters in columns mean statistical insignificance; otherwise, they mean statistical significance (*p* < 0.05)).

Numerous studies have shown that LPS can induce inflammation, leading to oxidative stress and mitochondrial dysfunction, as well as the induction of autophagy [23, 24]. Mitochondria play a vital role in this process, which secrete mtDNA and ROS to activate the inflammasome and induce autophagy. Reports have shown that the overgeneration of ROS exerted a huge impact on mitochondrial metabolism [25]. Additionally, mitochondrial dysfunction, characterized by the decrease of the mitochondria and the imbalance of the lipid peroxidation of the mitochondrial membrane (MMMP) and mitochondrial membrane potential (MMP), is correlative to oxidative stress caused by inflammation [26]. As shown in Figure 4, LPS treatment declined the mean fluorescence intensity of MitoTracker Green and NAO, while increasing the mean RH123 fluorescence intensity. However, these trends were suppressed by ALE, indicating that the reduction of mitochondria was inhibited after ALE pretreatment, while MMMP and MMP were significantly alleviated (Figures 4(b), 4(e), 5(c), and 5(f)). It has been reported that flavonoids can protect mitochondria from oxidative damage and remove excessive free radicals both *in vitro* and *in vivo*, which is consistent with our results [27, 28].

### 3.6. ALE Ameliorates Oxidative Stress and Inflammation via NF-*κ*B, Nrf2-Keap1, and MAPKs in RAW264.7 Cells

NF-*κ*B is an important factor related to the regulation of inflammatory mediators. The transport of NF-*κ*B is involved in the expression of iNOS and COX2 [28, 29]. The above studies have confirmed that ALE can significantly inhibit the overproduction of NO; thus, we further detect the effects of ALE on expressions of iNOS, COX2, and NF-*κ*B. The results showed that LPS upregulated the expressions of iNOS, COX2, and NF-*κ*B, while the treatment of ALE could suppress such upregulation triggered by LPS (Figures 5(a) and 5(c)–5(e)) thereby inhibiting the production of NO in cells. It has also been confirmed in above results that ALE could also attenuate the oxidative damage induced by LPS (Figure 3). As an essential protein in antioxidative stress, Nrf2 is able to help regulate the oxidative stress [30]. In order to investigate whether the anti-inflammatory effect of ALE is related to the Nrf2 pathway, we examined the expression of Nrf2 and Keap1 [30]. ALE significantly activated the expression of these two proteins in the presence of LPS, indicating that ALE promoted the dissociation of Nrf2/Keap1 and activated the Nrf2-mediated antioxidant cascade in RAW264.7 cells (Figures 5(a), 5(f), and 5(g)). MAPKs, including JNK1/2, ERK1/2, and p38 MAPK, are crucial in adjusting the inflammation induced by external factors [31]. ERK, which exists widely in various tissues, is involved in the regulation of cell proliferation and differentiation. JNK is a key in cell signal transduction induced by various factors and related to cell stress responses such as radiation, osmotic pressure, and temperature [32]. p38 is involved in inflammation and apoptosis [4]. The phosphorylation of MAPKs induced by LPS was involved in the activations of iNOS, COX2, and proinflammatory factors [32]. In order to confirm the effect of ALE on the expressions of MAPKs in RAW264.7 cells, we detected these proteins. Results showed that ALE inhibited the phosphorylation of MAPKs induced by LPS (Figures 5(b) and 5(h)–5(j)), indicating that ALE could relieve inflammation through MAPK pathways. The results above manifested that ALE ameliorated oxidative stress and inflammation via NF-*κ*B, Nrf2-Keap1, and MAPKs in RAW264.7 cells.

### 3.7. ALE Activates Autophagy to Resist Inflammation in RAW264.7 Cells

Redundant ROS/RNS will induce the mitochondrial dysfunction and the activation of mitochondrial autophagy against inflammation [31]. Autophagy is an intracellular self-protection mechanism that degrades misfolded proteins and dysfunctional organelles by lysosomes. Autophagy is involved in a variety of diseases including cancer, diabetes, aging, neurodegenerative diseases, inflammation, and liver diseases. In most cases, autophagy is both cytoprotective and destructive [32].

**Figure 6:**
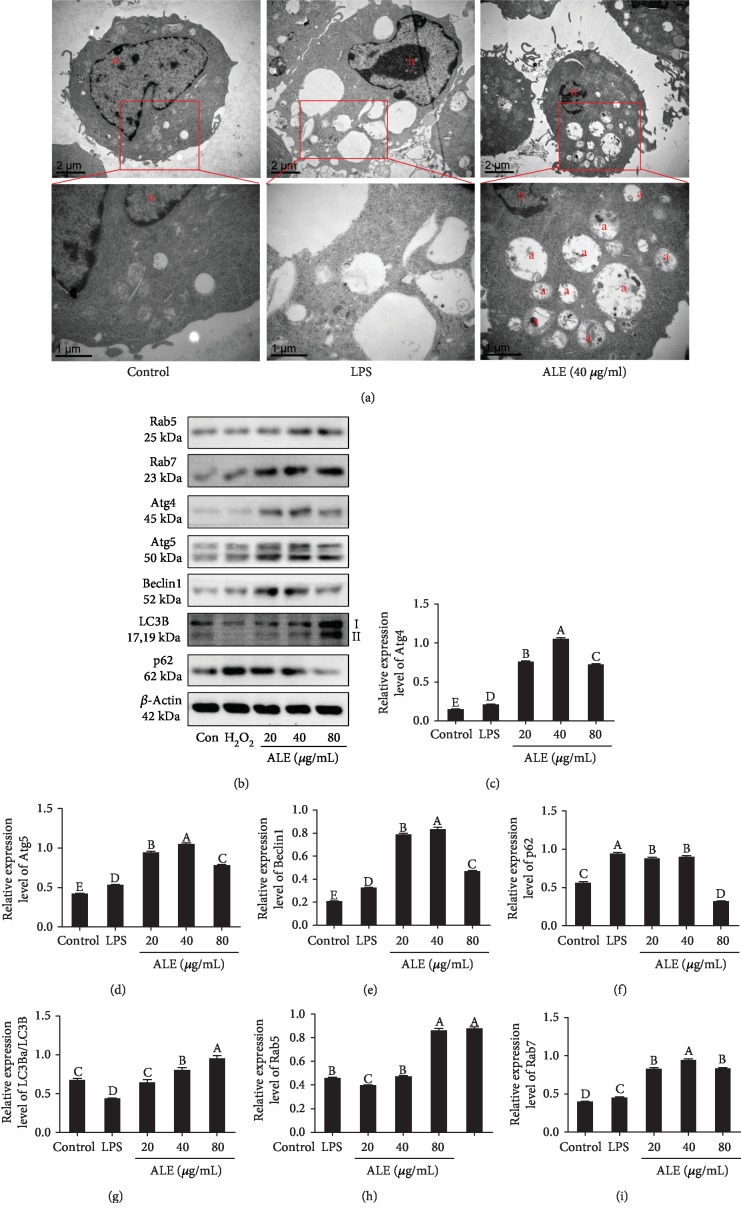
ALE activated autophagy in LPS-induced RAW264.7 cells. (a) Transmission electron microscopy features of macrophages (*n* = 3). (b) Western blot bands of Rab5, Rab7, Atg4, Atg5, Beclin1, LC3B, p62, and *β*-actin (*n* = 3). (c–i) Relative expression levels of Atg4, Atg5, Beclin1, p62, LC3BII/LC3BI, Rab5, and Rab7. The images were quantified by the ImageJ software, and the intensity of bands was corrected with *β*-actin (*n* = 3); vertical lines in the histogram represent SDs of three replicates. Significance analysis was carried out according to the one-way ANOVA test, and different letters mean statistically significant differences among the groups (A, B, C, D, and E were labeled from large to small and the same letters in columns mean statistical insignificance; otherwise, they mean statistical significance (*p* < 0.05)).

In our study, ALE could reduce oxidative stress, mitochondrial damage, and inflammation induced by LPS, which was consistent with previous studies [4]. So, we speculated that ALE could activate the autophagy in macrophages to resist inflammation. The result of TEM showed that the number of autophagosomes increased after ALE treatment, suggesting that ALE could enhance autophagy in RAW264.7 cells (Figure 6(a)). Western blot results showed that compared with the LPS-treated group, the expressions of Beclin1, Atg4, and Atg5 were upregulated in the ALE-treated group, while the expression level of p62 was significantly inhibited in a dose-dependent manner (Figures 6(c)–6(i)). Meanwhile, the expression of LC3II/LC3I was enhanced after the treatment of ALE. These results together confirmed that ALE could activate autophagy to resist inflammation in LPS-induced RAW264.7 cells [32].

### 3.8. ALE Induces Cell Autophagy via HMGB1, FoxO1, and Akt-mTOR Pathways in RAW264.7 Cells

**Figure 7:**
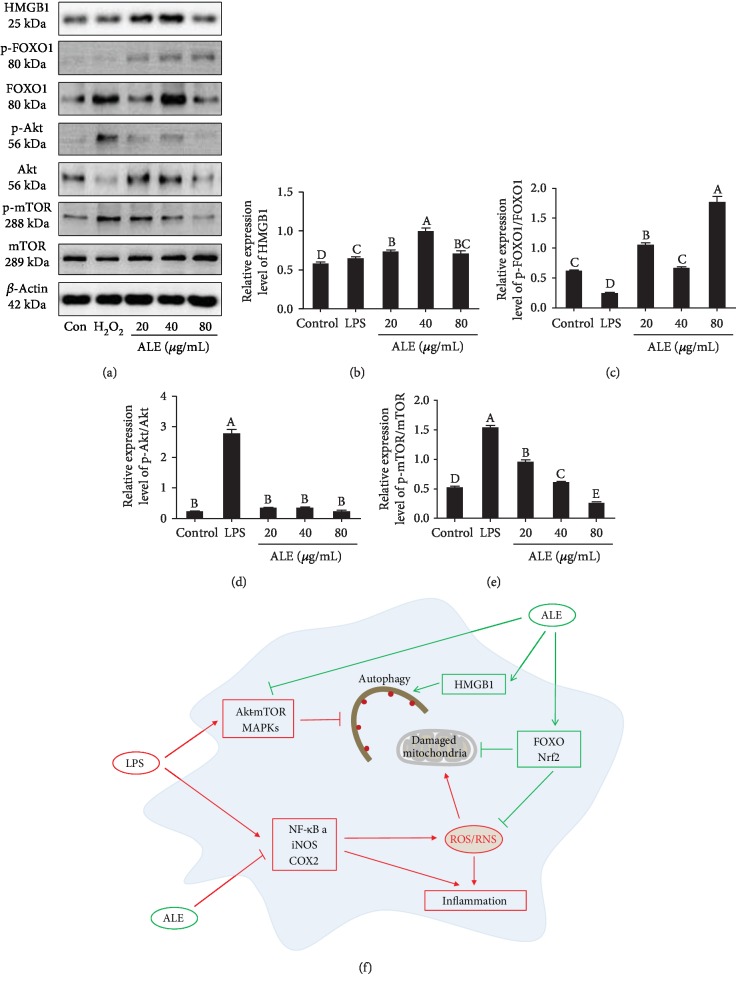
Effect of ALE on expression levels of HMGB1, FoxO1, and Akt-mTOR pathways in RAW264.7 cells. (a) Western blot bands of HMGB1, p-FOXO1, FOXO1, p-Akt, Akt, p-mTOR, mToR, and *β*-actin (*n* = 3). (b–e) Relative expression levels of HMGB1, p-FOXO1/FOXO1, p-Akt/Akt, and p-mTOR/mToR. The images were quantified by the ImageJ software, and the intensity of bands was corrected with *β*-actin (*n* = 3); vertical lines in the histogram represent SDs of three replicates. (f) The schematic of the mechanism of impact of ALE on LPS-induced RAW264.7 cells. Significance analysis was carried out according to the one-way ANOVA test, and different letters mean statistically significant differences among the groups (A, B, C, D, and E were labeled from large to small and the same letters in columns mean statistical insignificance; otherwise, they mean statistical significance (*p* < 0.05)).

ALE can induce autophagy, which facilitates in reducing inflammation. There are many signal pathways concerned with the regulation of autophagy as previously reported [33]. HMGB1, FoxO1, and Akt-mTOR are the key regulators to control autophagy [22, 34–36]. Researchers have proven that HMGB1 facilitates the phosphorylation of Bcl-2 to promote the dissociation of Beclin1 from the Beclinl-Bcl2 compound and the combination to the autophagy regulator Beclin1, thereby contributing to the assembly of the PI3KC3-Beclin1 compound and autophagy [37]. We found that ALE triggered the expression of HMGB1 (Figures 7(a) and 7(b)) and the delivery of Beclin1 (Figures 6(b)–6(i)). Under stress, FoxO1 could combine with the promoter of Atg12, which led to the activation of autophagy. Our research confirmed that ALE contributed to the phosphorylation of FoxO1 and promoted the expression of Rab5 and Rab7 (Figures 6(b)–6(i)), suggesting that ALE played an important role in the fusion of autophagosomes and lysosomes. In addition, the Akt-mTOR pathway is important in cellular inflammation and autophagy. The intervention of ALE caused the downregulation of relative expressions of p-Akt/Akt and p-mTOR/mTOR (Figure 7(c)), thereby contributing to autophagy, which was shown in the results of western blot. In conclusion, ALE induces cell autophagy via activation of HMGB1 and FoxO1 and inhibition of Akt-mTOR pathways in RAW264.7 cells.

## 4. Discussion

Flavonoids from natural plants have attracted extensive attention due to their excellent biological activities (such as antioxidant, anti-inflammatory, antitumor, and antibacterial). It is generally accepted that plant-derived functional factors are nontoxic and have side effects [38]. Many studies have shown that the biological activities of flavonoids depend on their structural characteristics, and different flavonoids have different activities [39]. A large number of studies have shown that natural flavonoids have good anti-inflammatory activities. For example, apigenin can prevent colon inflammation and motor dysfunction in obese mice induced by a high-fat diet [40]. Naringin can reduce the cell viability, fat content, and inflammatory response of breast tumor cells in ovariectomized obese mice [41]. Liquiritigenin can reduce pulmonary inflammation and incidence of viral infection in mice [42]. The citrus flavonoid hesperidin can alleviate testicular injury in diabetic rats by inhibiting oxidative stress, inflammation, and apoptosis [43]. Baicalin can regulate the expression of inflammation-related genes in vivo to relieve skin inflammation [44].

In this study, a series of flavonoids were identified in ALE and the anti-inflammation effect of ALE was explored. Furthermore, ALE altered NF-*κ*B, MAPK, and Nrf2-Keap1 pathways to activate the anti-inflammatory system in RAW264.7 cells, at the same time via activating HMGB1-Beclin1 and FoxO1 pathways and inhibiting Akt-mTOR signaling pathways to induce cell autophagy, which could degrade damaged mitochondria and macromolecular substances, thereby lowering the secretion of NO and inflammatory cytokines. The anti-inflammatory processes of ALE involved in NF-*κ*B, MAPK, Nrf2-keap1, HMGB1-Beclin1, FoxO1, and Akt-mTOR signaling pathways and multiple pathways were linked to resist lipopolysaccharide-induced inflammatory response (Figure 7(e)).

During the anti-inflammation process, autophagy plays a vital role. Autophagy is an intracellular self-protective mechanism that degrades misfolded proteins and dysfunctional organelles through lysosomes [45]. Autophagy is also involved in a variety of diseases, including cancer, diabetes, aging, neurodegenerative diseases, inflammation, and liver diseases [46]. In most cases, autophagy is both cell protective and destructive. Numerous studies have shown that stimulation of LPS can induce inflammatory responses, leading to oxidative stress and mitochondrial dysfunction, as well as inducing autophagy. Mitochondria play a vital role in this process [47]. The secretion of mtDNA and ROS from mitochondria activates the inflammasome and induces impaired autophagy clearance, which is essential for activating the inflammatory response. In addition, oxidative stress can accelerate the formation of autophagy, and autophagy can reduce oxidative damage by degrading oxidative substances and damaged mitochondria [48, 49]. Our results confirm that ALE can induce autophagy of macrophages by activating the Hmgb1-Beclin1 and Sirt1-FOXO1 pathway and inhibiting the Akt-mTOR signaling pathway, thereby reducing LPS-induced oxidative stress, mitochondrial dysfunction, and inflammation in RAW264.7 cells. However, it is still unclear whether other pathways are also involved in regulating autophagy, and more studies are needed to demonstrate the interaction between ALE and autophagy in LPS-induced RAW264.7 cells.

## 5. Conclusion

Flavonoids derived from natural sources play significant roles in anti-inflammatory, antitumor, and antioxidant processes [50]. In this study, seven compounds were extracted from *Apios americana* Medik leaves and identified by LC-TOF-MS. Using *in vitro* studies, we first demonstrated that ALE could protect RAW264.7 cells from cytotoxicity, inflammation, oxidative damage, and mitochondrial dysfunction against LPS. Meanwhile, ALE reduced the overproduction of NO and inflammatory cytokines via inhibition of NF-*κ*B and MAPK signaling and activation of Nrf2-Keap1 and FOXO pathways [51]. In addition, ALE could activate HMGB1-Beclin1 and Sirt1-FoxO1 pathways and inhibit the Akt-mTOR signaling pathway to induce autophagy in RAW264.7 cells, thereby contributing to the decomposition of damaged mitochondria and maintaining ROS homeostasis. However, it is unclear whether other pathways are involved in the regulation of autophagy activation, and further research is needed to demonstrate the interaction between the protection effect of ALE and autophagy in RAW264.7 cells. Overall, this finding manifests that the leaf of *Apios americana* Medik has potential to be the source of an antioxidant and anti-inflammatory drug, which means *Apios americana* Medik leaves can be applied to promote human health in future.

## Figures and Tables

**Table 1:** Identification of ALE.

No.	RT (min)	Molecular ion (M+)	MS/MS (M+)	Tentative identification
1	20.063	593.1555	297.0759, 325.0710, 353.0671, 383.0772, 473.1107, 503.1217	Vicenin-2
2	21.737	563.1427	297.0763, 325.0713, 353.0668, 383.0776, 413.0880, 443.0995, 473.1106	Schaftoside
3	22.033	625.1432	151.0029, 178.9978, 271.0238, 300.0272, 301.0343, 343.0454	Baimaside
4	22.217	563.1422	149.0240, 191.0342, 221.0454, 297.0770, 353.0668, 383.0776, 413.0878, 433.0986, 473.1100, 545.1307	Apigenin (6-C-*α*-L-arabinopyranosyl)-8-C-*β*-D-glucopyranoside
5	23.653	431.0994	161.0238, 268.0368, 281.0449, 283.0607, 311.0557, 341.0667	Vitexin
6	24.133	463.0883	151.0030, 243.0296, 255.0293, 271.0247, 300.0265, 301.0345	Isquercetin
7	24.397	461.1116	297.0397, 298.0478, 341.0674, 371.07788, 461.1116	6-C-*β*-D-Glucopyranosyl-7-O-methylluteolin

## Data Availability

The data used to support the findings of this study are available from the corresponding author upon request.
